# Using the health belief model to understand the factors influencing the perceptions of people of Chinese ancestry about reducing salt consumption for hypertension prevention: A cross-sectional study

**DOI:** 10.1371/journal.pone.0289867

**Published:** 2023-08-16

**Authors:** Alex Chan, Sally Wai-chi Chan, Leigh Kinsman

**Affiliations:** 1 School of Nursing and Midwifery, The University of Newcastle, Newcastle, Australia; 2 School of Nursing, University of Wollongong, Wollongong, Australia; 3 Tung Wah College, Hong Kong SAR, China; 4 La Trobe Rural Health School, La Trobe University, Bendigo, Australia; Zagazig University Faculty of Human Medicine, EGYPT

## Abstract

**Background:**

High-salt diets are linked to hypertension. Chinese people in Australia, are at increased risk of hypertension due to the combination of routine addition of high quantities of salt to food during cooking and high salt levels in processed western foods. There is a scarcity of salt-related behavioural studies on this population group. This study aimed to explore the habitual salt consumption of Chinese Australians and factors that influence their perceptions about sustaining salt-related behavioural changes for hypertension prevention.

**Method:**

A cross-sectional descriptive study using an adapted Determinants of Salt-Restriction Behaviour Questionnaire was conducted on 188 Chinese Australians. A non-probability sampling method was used to attract participants from different parts of Australia. Statistical analyses such as descriptive analysis, t-tests and Pearson correlation tests were performed in the study.

**Results:**

Over 97% of participants did not measure the amount of salt added to their meals. Many participants reported that salt was added to their meals based on their experience (39.4%) and food taste (31.9%). Over 80% of participants did not know the recommended level of daily salt consumption. Although salt-related knowledge had no significant correlation with individuals’ salty food taste preferences, there were significant correlations with the perceptions of the severity of disease and health benefits of reducing salt consumption (p = .001 and < .001 respectively). People with stronger salty taste preferences perceived a higher level of health threat than people with lighter salty taste preferences (p = .003).

**Conclusion:**

Findings from this study show that knowledge about salt-reduction alone had no significant effects on salt-related behaviours. Adequate culturally relevant practice-based education in salt-reduction strategies may facilitate salt-related behavioural changes in Chinese Australians. Overall, single young Chinese Australian men with stronger salty taste preferences is the group who needs salt reduction interventions the most.

## Introduction

High dietary salt consumption is a threat to global health, and dietary salt-reduction is an effective measure for lowering the risk of hypertension, a modifiable cardiovascular disease risk factor [1–3]. Dietary salt-reduction is an effective self-management intervention for hypertension prevention and reduces the overall risk of hypertension-related cardiovascular diseases such as heart failure and stroke [4]. Currently, the World Health Organization (WHO) recommends restricting dietary salt consumption to less than 5 grams per day (g/day) for healthy adults [5]. However, previous studies found that many adults around the globe, especially those from some ethnic groups, consumed a significantly higher amount of salt every day. For example, on average, Australians consumed 9.6 g/day in 2015 [6], Italians 9.0 g/day between 2008 and 2012 [7], Russians 11.35g/day in 2018 [8] and Chinese in Mainland China more than 10 g/day in 2018 [9].

In general, salt intake that is acquired from discretionary sources, with salt added during cooking or at the table, is a growing health concern [10]. A systematic review found that people in China, Japan and India consumed more than half of their total daily salt intake from discretionary sources, compared with less than a quarter of daily salt intake among people in Australia, the United Kingdom (UK) and Denmark [10].

Australia is a popular destination for immigrants. In 2020, over 7.6 million migrants lived in Australia, and 29.8% of Australian residents were born overseas [11]. China has been one of the largest sources of migrants to Australia since 2011 [12]. The 2016 census found that more than 1.2 million people of Chinese ancestry resided in Australia, comprising nearly 5% of the entire Australian population [13]. According to the Australian Bureau of Statistics, Chinese ancestry refers to people who have the similarity of Chinese cultural and ethnic group in terms of social and cultural characteristics [14]. In this study, Chinese Australians refer to residents of Chinese ancestry in Australia.

Hypertension is a very serious health problem in China [15]. It was estimated that 1 in 4 Chinese adults suffered from hypertension in 2012–2015 [15]. For Chinese Australians there is a risk of even higher salt intake through the combination of discretionary sources and greater exposure to processed foods in a western country, particularly as 75% of Chinese Australians were first-generation Australian residents [13]. The effects of socialisation and post-migration lifestyle changes may alter migrants’ dietary habits and attitudes, leading to an increase or decrease in their overall salt intake.

### Health belief model

According to the Health Belief Model (HBM), individuals are likely to initiate a health behavioural change if they perceive themselves at risk of a specific health condition such as hypertension and believe that this condition would have a significant negative impact on their health [16]. In order to sustain health behavioural changes, people must believe that the benefits are greater than the barriers they have to overcome and that they are capable of making the change [16, 17]. A recent literature review [18] indicated that the level of educational exposure to the health benefits of salt-reduction, simple salt measurement methods and Chinese dietary culture and practice significantly affected the adoption of salt-reduction in Chinese people.

Based on the HBM and findings in other salt-related behavioural studies in Chinese populations, gender, BMI, salty food taste preferences, relationship status, history of hypertension, routine salt measurement, doctors’ advice, families’ and friends’ advice and media advertising may influence individuals’ perceptions about sustaining salt-related health behavioural changes [19, 20]. However, these factors have yet to be investigated in the Chinese Australian context. This is an important issue as these factors may have a profound effect on the desire among Chinese Australians to reduce salt consumption.

There is a paucity of studies conducted on dietary salt consumption among minority population groups such as people of Chinese ancestry in western countries [20–22], with studies focused on the main ethnic groups of the countries [23, 24]. It is important to note that dietary practice may be influenced not only by personal preferences and cultural inheritance, but also by a range of miscellaneous factors, such as the availability of culturally appropriate health education, peer support networks where people share knowledge and support each other to encourage healthy behaviours [25] and affordability of healthy and sustainable diets [26]. In summary, Chinese Australians especially the first generations are likely to continue their inherent dietary practice and may selectively westernise their diet in Australia. The salt-related health risks may remain or even higher than their counterparts in China. To our knowledge, there is no specific culturally appropriate salt-reduction health champion for this group of population in Australia.

The aim of the study was to explore the habitual salt consumption in people of Chinese ancestry in Australia (Chinese Australians), including factors that influence their perceptions about reducing average salt consumption for the prevention of hypertension. A convenience sample and the adapted Determinants of Salt-Restriction Behaviour Questionnaire (DSRBQ) [27] were used to conduct the study over a period of 14 months.

The study findings could not only assist nurses and other health care providers to tailor existing salt-reduction risk management strategies for hypertension in this population, but also increase the representation of this ethnic minority group in Australian preventive health research.

## Methods

### Study design

A cross-sectional descriptive survey was conducted among Chinese Australians. Online and paper-based self-administered DSRBQs in either the English or Chinese language were used to collect data. The STrengthening the Reporting of OBservational studies in Epidemiology (STROBE) checklist for cross-sectional studies was used to guide the preparation of this report [28].

### Setting and participants

The study was conducted in Australia from January to March 2020 and then from July 2020 to May 2021. Data collection was suspended between March and July 2020 due to the coronavirus (COVID-19) pandemic in Australia, when people were overwhelmed by COVID-19-related health information and lockdowns. A convenience sample was recruited from social media. The inclusion criteria were as follows: a) adults over 18 years old; those of Chinese ancestry; and b) those who had lived in Australia for at least 6 months. Adults who were unable to read a Chinese or English questionnaire were not included. In the 2016 Census, there were approximately 1.2 million people identified as having Chinese ancestry in Australia [29]. The sample size was calculated using the SurveyMonkey’s online sample size calculator [30], which was based on the following calculation formula [31]:

$$\text{Sample}\;\text{size}=\left[\text{p}\left(1-\text{p}\right)\times {\text{z}}^{2}/{\text{e}}^{2}\right]/\left[1+\text{p}\left(1-\text{p}\right)\times {\text{z}}^{2}/{\text{ne}}^{2}\right]$$

*Abbreviations*: *n* = *population size; p* = *population proportion; e* = *margin of error; z* = *z-score*.

The acceptable margin of error generally ranges from 1% to 10% at the 95% confidence level [31]. In general, the sample size decreases while the margin of error increases [31]. Given that data collection was conducted during the COVID-19 pandemic when people had different health priorities, a margin of error of 8% was used in this study. It is important to acknowledge that using a higher margin of error to calculate the sample size may increase the risks of types I and II statistical errors, with the result that a null hypothesis is falsely rejected or accepted [32]. To achieve a margin of error of 8% at the 95% confidence level, the target sample size was a minimum of 151 participants. Considering the key objectives of the study were not to test a range of hypotheses, researchers believed a minimum of 151 participants in the sample would be sufficient.

A recruitment flyer with the survey link was posted on social media such as Facebook, WeChat, Weibo and two Chinese community radio programs’ social media pages. Those who were interested in participating accessed the survey link online, read the participant information sheet, and completed the survey. People who preferred to complete a paper-based questionnaire contacted the researcher (first author of the paper) by email. A participant information statement and a reply-paid envelope were sent to them in a post pack. The participants completed the questionnaire online or via the paper-based version, with the choice of either Chinese or English language.

### Instrument

The DSRBQ was developed in Beijing, China and has been used previously in three studies, n = 513 [33], n = 403 [34] and n = 799 [35]. This questionnaire was based on the HBM, a psychological framework that explains and predicts changes in health behaviours [36]. The authors translated the original DSRBQ into English and the adapted versions were validated within the Chinese Australia context [27]. Both adapted Chinese and translated English versions had good reliability, with Cronbach’s alpha scores of 0.638 and 0.584 respectively, and overall intra-class correlation coefficients of 0.820 and 0.688 respectively. The adapted DSRBQ consists of 3 parts with a total of 73 items, requiring approximately 30 minutes to complete (Table 1).

**Table 1:** Descriptions of the adopted determinants of salt-restriction behaviour questionnaire.

**Part 1**	A total of 22 items: Demographic characteristics (n = 9), including age, gender, place of birth, education, marital status, employment, income and health conditions. Personal dietary practice (n = 9): 3 items measured using categorical variable scales 3 items measured using 5-point Likert scales ranging from ‘daily (1) to never (5)’, ‘never (1) to always (5)’ and ‘very light (1) to very salty (5)’ 2 items requiring participants to directly answer the number of meals being consumed at home and the percentage of food consumed at home vs away from home. 1 binary scale (yes/no) items. Salt-related health education/medical advice that the participant has received (n = 4), measured using a binary scale (yes/no).
**Part 2**	Salt-related health knowledge (n = 6) measured using categorical variable scales.
**Part 3**	A total of 45 items forming six subscales: Perceived threat (n = 5) Knowledge/perceived susceptibility to and severity of the disease (n = 6) Perceived benefits of action (n = 3) Perceived benefits of using a measuring spoon (n = 3) Likelihood of following the recommended interventions (n = 10) Perceived barriers (n = 18). All items are measured using a 5-point Likert scale ranging from strongly disagree (1) to strongly agree (5).

### Measures

#### Sociodemographic and dietary practice characteristics

Sociodemographic data such as age, gender, place of birth, education, relationship status, employment, weight, height, income and past medical history were collected in part 1 of the questionnaire. Participants’ dietary practice was measured through nine items, including how often they ate at home, food seasonings and salt usage, salty food taste preferences, and if they used a measuring spoon to limit salt being added to foods. The salty food taste preferences were measured through a slider rating scale. The respondents moved a horizontal slider to rate their food taste preferences on a scale of 0 (very light) to 100 (very salty).

#### Salt-related health knowledge

Salt-related health knowledge was assessed in part 2 by 6 categorical questions about the WHO-recommended maximum daily salt consumption level [4], long-term health issues associated with a high-salt diet, diagnostic criteria for hypertension, and causes, complications and preventive strategies for hypertension. One point was awarded to each correct answer [37]. This section had a maximum score of 6 points.

#### Individuals’ perceptions about sustaining salt-related behavioural changes

Perceptions about sustaining behavioural changes (salt-reduction) were measured in part 3 through a series of Likert Scale items (n = 45). Part 3 was composed of six subscales: perceived health threats (n = 5); severity of the disease and susceptibility (n = 6); benefits of action (n = 3); barriers to reducing salt intake (n = 18); benefits of using a measuring spoon (n = 3); and likelihood of following the recommended interventions (n = 10). Participants responded on a 5-point Likert scale ranging from strongly disagree (1) to strongly agree (5).

### Data analysis

Descriptive statistics such as means and percentages were used to summarise the demographics (age, gender, place of birth, education, relationship status, employment, income and health conditions). The Pearson correlation coefficient (*r*) test was used to examine associations between continuous variables [38] such as age, BMI and salt-related behaviours, attitudes towards salt-reduction, and barriers to reducing salt consumption. To measure differences between variables, independent t-tests (t) were used for binary and continuous variables [38]. The confidence interval (CI) percentage was set at 95% when computing the t-tests. A p-value less than .05 was considered to be statistically significant. SPSS software version 25 was used for all analyses (IBM Corp. Released 2017. IBM SPSS Statistics for Windows, Version 25.0. Armonk, NY: IBM Corp.). To reduce the risk of bias in the statistical analysis, participants with missing items that constituted more than 10% of the questionnaire were excluded from data analysis [39].

### Ethical considerations

Ethics approval was granted by the Human Research Ethics Committee at the University of Newcastle, Australia in which the study was conducted (approval number: H-2019-0180), and permission to use the questionnaire was obtained from the author of the DSRBQ [35]. Informed consent to participate was assumed by participants, and no identifiable information such as name and home address was requested. Before completing the anonymous questionnaire, participants could choose to receive a paper copy or electronic copy of the participant information statement, which detailed the purpose and aims of the study. Participants were asked to confirm consent to the study by ticking a box if they were willing to proceed. Participation was voluntary. As the questionnaire was anonymous, participants were unable to withdraw from the study once their responses were submitted. On the completion of the questionnaire, if participants chose to be included in a draw to win one of three $100 gift vouchers, they were asked to enter their contact details in a separate database so that their responses could not be identified.

## Results

**Fig 1:**
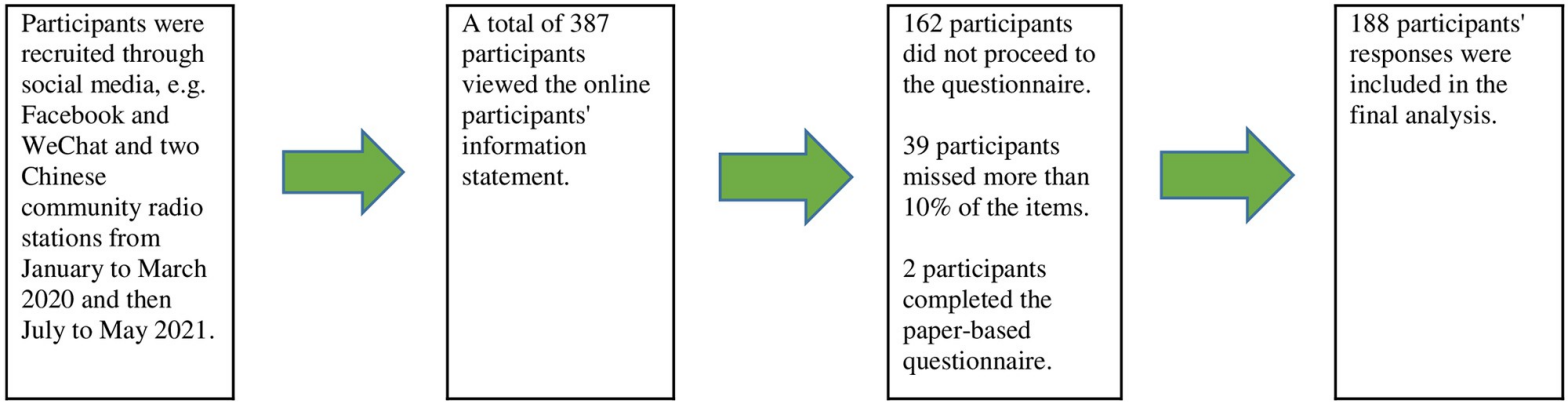
Recruitment process.

A total of 389 participants read the online participants’ information statement. One hundred and sixty-two (162) participants did not proceed to the questionnaire. Thirty-nine (39) participants’ responses were excluded from the study because they had missed more than 10% of the items. Two (2) participants completed a paper-based questionnaire. As a result, the total sample included 188 participants (Fig 1).

### Participant characteristics

The socio-demographic characteristics of the participants were presented in Table 2. Women comprised 64.9% (n = 122) of the sample. The sample’s age ranged from 19 to 79 (mean = 36, SD = 10.9). The mean BMI was 23.20 (SD = 4.12), and 44.7% (n = 84) of participants’ BMI was within the normal Asia-Pacific BMI range, 18.5–22.9 [40]. 162 participants (86.2%) reported no medical history of hypertension. The age of people with hypertension (n = 14) ranged from 24 to 79 years (mean = 36.0, SD = 11.9). Of these, 12 participants (85.7%) had a higher BMI (>23.0). The independent t-test demonstrated that participants with hypertension had a statistically higher BMI than participants with normotension (t = 4.603, p = < .001, 95% CI: 3.313–9.074).

**Table 2:** Demographic characteristics of the participants.

Characteristics	N = 188
**Gender**	
***Male***	64 (34%)
***Female***	122 (64.9%)
***No response***	2 (1.1%)
**Age: mean (SD) [median]**	36 (10.9) [34]
**Hypertension**	
***Diagnosed hypertension***	14 (7.4%)
***Normotension***	162 (86.2%)
***Unknown***	12 (6.4%)
**Marital status**	
***Married***	79 (42.0%)
***Single/separated/divorced/widowed***	109 (58.0%)
**Place of birth**	
***Greater China Region***:	
• ***Mainland China***	63 (33.5%)
• ***Hong Kong***	69 (36.7%)
• ***Taiwan***	26 (13.8%)
***Australia***	10 (5.3%)
***Others–e***.***g***. ***Malaysia***, ***Singapore***.	20 (10.7%)
**Education level**	
***Primary***	3 (1.6%)
***Secondary***	20 (10.7%)
***Tertiary or university***	164 (87.2%)
***No response***	1 (0.5%)
**Occupation**	
***Professional***	14 (7.4%)
***Semi-professional***	9 (4.8%)
***Non-professional***	70 (37.2%)
***Self-employed***	15 (8.0%)
***Not in workforce***, ***e***.***g***. ***full-time mothers and students***	67 (35.6%)
***Retired***	11 (5.9%)
***No response***	2 (1.1%)
**Household income (per annum in Australian dollars)**	
***<$20***,***800***	14 (7.4%)
***$20***,***800-–$41***,***599***	21 (11.2%)
***$41***,***600–$64***,***999***	47 (25.0%)
***$65***,***000–$103***,***999***	40 (21.3%)
***≥$104***,***000***	64 (34.0%)
***No response***	2 (1.1%)

Data were collected from a total of 188 participants. Values were presented as numbers and percentages (N and %).

Most participants originated from the Greater China Region: Hong Kong (n = 69, 36.7%), China (n = 63, 33.5%) and Taiwan (n = 26, 13.8%), 10 participants (5.3%) were born in Australia, and the remaining were born in other countries including the UK.

In 2019–2020, the median gross household income in Australia was AU$92,872 (US$62,251) [41]. Within our sample, 104 participants (55.3%) reported household incomes above $65,000 (US$43,611), and of these, 64 participants (34.0%) reported household incomes above $104,000 (US$69,778) per annum, which was above the national median household income.

### Habitual salt consumption behaviours

**Fig 2:**
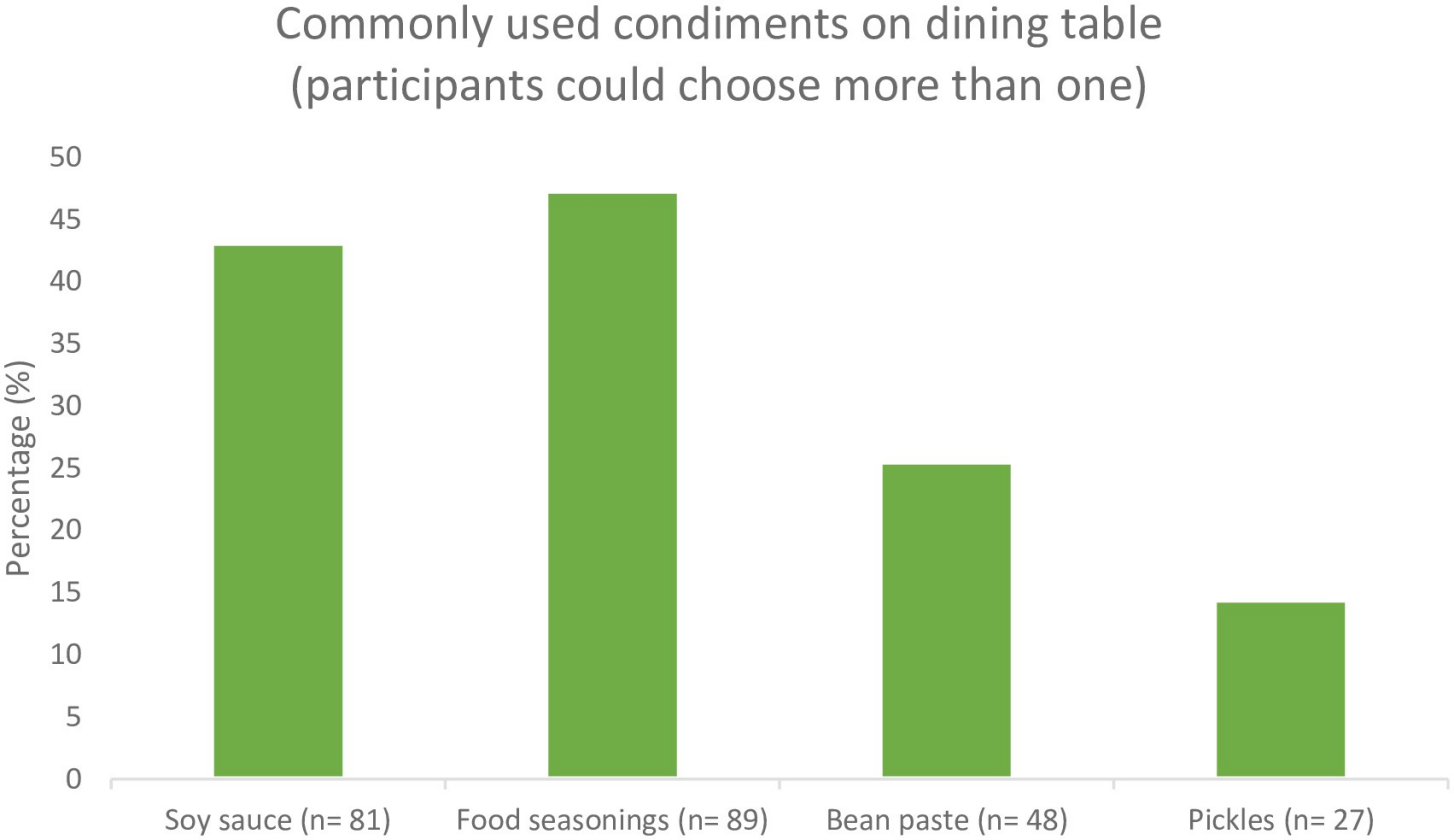
Commonly used condiments.

More than 53% (n = 101) of the participants reported they often added high-salt condiments such as soy sauce (n = 81, 43.1%), food seasonings (n = 89, 47.3%), bean paste (n = 48, 25.5%) and pickles (n = 27, 14.4%) to food on the dining table (Fig 2). Over 60% (mean = 60.6%, SD = 21.3) of food consumption occurred at home. A small number of participants (n = 29, 15.4%) reported they had a measuring spoon, but only 5 (2.7%) participants measured and followed the recommended daily salt intake when they added salt to their meals during cooking. Many participants determined the amount of salt being added to their meals based on their experience (n = 74, 39.4%) and preferred taste (n = 60, 31.9%) (Table 3).

**Table 3:** Habitual salt consumption behaviours.

	N (%)
**Follow the recommended daily salt consumption**	5 (2.7%)
**Follow their experience**	74 (39.4%)
**Follow their preferred taste**	60 (31.9%)
**Never use a measuring spoon to measure cooking salt**	37 (19.7%)
**Others, e.g. when following a recipe to cook and a pinch of salt is part of the recipe**	11 (5.9%)
**No response**	1 (0.5%)

Data were collected from a total of 188 participants. Values were presented as numbers and percentages (N and %).

### Salt-related health knowledge

The mean score of the salt-related health knowledge items in part 2 was 4.26 (SD = 1.04). A total of 168 participants (89.4%) indicated that had never received medical advice on salt restriction. Half of the participants (n = 94, 50%) were aware of the link between long-term high dietary salt consumption and hypertension. Moreover, awareness of renal diseases associated with long-term high dietary salt consumption was 35.6% (n = 67). Sixteen participants (8.5%) indicated that they were unaware of the impact of excessive salt intake on their health. The findings in relation to participants’ salt-related knowledge were presented in Table 4. Overall, there was no significant difference among participants with normotension or hypertension with regard to their perception of high salt consumption being a health risk (t = 1.465, p = .145, 95% CI: -.039 –.265) and their level of salt-related health knowledge (t = -.216, p = .829, 95% CI: -.642 –.515). Women had significant higher level of salt-related health knowledge than men (t = -1.983, p = .049, 95% CI: -.628 –-.002).

**Table 4:** Salt-related knowledge items (part 2).

Salt-related knowledge items	Correct N (%)	Incorrect N (%)	Don’t know N (%)	Non-response N (%)
**Q1—The WHO-recommended maximum daily salt consumption level**.	28 (14.9%)	53 (28.2%)	106 (56.4%)	1 (0.5%)
**Q2—The long-term effects of high-salt dietary intake**.	169 (89.9%)	3 (1.6%)	16 (8.5%)	nil
**Q3—The diagnostic criteria for high blood pressure**.	71 (37.8%)	63 (33.5%)	53 (28.2%)	1 (0.5%)
**Q4—The cause(s) of hypertension**.	180 (95.7%)	2 (1.06%)	6 (3.2%)	nil
**Q5—Selected the ‘low salt diet’ option as a high blood pressure prevention measure**.	165 (87.8%)			nil
**Q6—Selected ‘don’t know’ the complications of poor blood pressure control**.			14 (7.4%)	nil

Data were collected from a total of 188 participants. Values were presented as numbers and percentages (N and %).

### Factors affecting perceptions about sustaining salt-related behavioural changes

The correlations between salt-related knowledge, age, BMI and salty taste preferences with perceptions about sustaining salt-related behavioural change subscales were summarised in Table 5. Individuals’ salty taste preferences positively correlated with one of the subscales (perceived health threats). The stronger the salty taste preferences, the higher the level of perception of the health threats (r = .215, p = .003), and participants experienced higher barriers to reducing their salt consumption (r = .415, p = < .001).

**Table 5:** Correlations between salt-related knowledge, age, body mass index, salty taste preferences and the subscales related to perceptions of sustaining salt-related behavioural changes (Pearson Correlation tests).

	Perceived health threats	Perceived severity and susceptibility of disease	Perceived benefits of taking action	Perceived benefits of using a measuring spoon	Likelihood of following the recommended interventions	Perceived barriers to reducing salt intake	Salty taste preferences
**Salt-related knowledge**	*r* = .112	*r* = .232	*r* = .251	*r* = .189	*r* = .079	*r* = -.099	*r* = -.088
	p = .124	p = .001*	p = < .001*	p = .009*	p = .284	p = .177	p = .228
**Age**	*r* = .061	*r* = -.021	*r* = .030	*r* = .009	*r* = -.058	*r* = .026	*r* = -.152
	p = .403	p = .774	p = .685	p = .900	p = .426	p = .728	p = .038*
**Body mass index**	*r* = .139	*r* = .088	*r* = -.137	*r* = -.063	*r* = -.110	*r =* .107	*r =* .013
	p = .058	p = .232	p = .062	p = .393	p = .134	p = .142	p = .857
**Salty taste preferences**	*r* = .215	*r* = -.028	*r* = -.028	*r* = -.045	*r* = -.178	*r* = .415	
	p = .003*	p = .708	p = .702	p = .542	p = .015*	p = < .001*	

Here, *r* = Pearson Correlation; p = probability value; and

* = statistically significant result.

A p-value of <0.05 is considered statistically significant.

Two significant weak negative correlations were found among salty taste preferences and the likelihood of following the recommended interventions and age: the stronger the salty taste preferences, the lower the likelihood of following the recommended interventions (r = -.178, p = .015) and the younger the age (r = -.152, p = .038).

The differences between key factors and perceptions about sustaining salt-related behavioural change subscales were reported in Table 6. The independent t-test demonstrated that compared with women, men perceived greater barriers to reducing salt intake (t = 3.111, p = .002, 95% CI: .083 –.370). Participants who were single had significantly higher perceived barriers to reducing salt consumption than married participants (t = 2.174, p = .031, 95% CI: .014 –.301). In relation to salty food taste preferences, single participants preferred to consume saltier foods than married participants (t = 2.392, p = .018, 95% CI: .114–1.184).

**Table 6:** Differences between key factors and the subscales of the perceptions related to sustaining salt-related behavioural changes (t-tests).

95% CI	Perceived health treats (Mean)	Perceived severity of disease (Mean)	Perceived benefits of taking action (Mean)	Perceived benefits of using a measuring spoon (Mean)	Likelihood of following the recommended interventions (Mean)	Perceived barriers to reducing salt intake (Mean)	Salty food taste preferences (Mean)
**Likert Scale**	Strongly disagree (1) to strongly agree (5)						Very light (1) to very salty (10)
**Gender**	Male = 3.25	Male = 3.88	Male = 3.80	Male = 3.50	Male = 3.25	Male = 3.01	Male = 4.53
	Female = 3.19	Female = 3.97	Female = 3.99	Female = 3.70	Female = 3.40	Female = 2.78	Female = 4.33
	t = 1.561	t = -.896	t = -1.636	t = -1.644	t = -1.691	t = 3.111	t = .683
	p = .120	p = .372	p = .105	p = .103	p = .093	p = .002*	p = .495
	CI = -.017, .148	CI = -.272, .103	CI = -.431, .041	CI = -.452, .042	CI = -.323, .025	CI = .083, .370	CI = -.372, .767
**Marital status**	Single = 3.23	Single = 3.91	Single = 3.87	Single = 3.62	Single = 3.35	Single = 2.92	Single = 4.68
	Married = 3.17	Married = 3.97	Married = 3.98	Married = 3.63	Married = 3.31	Married = 2.77	Married = 4.03
	t = 1.356	t = -.646	t = -1.046	t = -.072	t = .475	t = 2.174	t = 2.392
	p = .177	p = .519	p = .297	p = .943	p = .636	p = .031*	p = .018*
	CI = -.025, .136	CI = -.227, .115	CI = -.322, .099	CI = -.221, .206	CI = -.128, .210	CI = .014, .301	CI = .114, 1.184
**History of hypertension**	HT = 3.31	HT = 3.77	HT = 3.38	HT = 3.29	HT = 3.11	HT = 3.06	HT = 4.00
	NT = 3.20	NT = 3.96	NT = 3.99	NT = 3.67	NT = 3.39	NT = 2.83	NT = 4.37
	t = 1.465	t = -1.096	t = -1.983	t = -1.174	t = -1.735	t = 1.707	t = -.722
	p = .145	p = .274	p = .068	p = .260	p = .085	p = .090	p = .471
	CI = -.039, .265	CI = -.507, .145	CI = -1.259, .050	CI = -1.095, .321	CI = -.586, .038	CI = -.036, .500	CI = -1.397, .648
**Salt measurement**	Yes = 3.25	Yes = 3.60	Yes = 3.87	Yes = 3.60	Yes = 3.44	Yes = 3.00	Yes = 4.60
	No = 3.20	No = 3.94	No = 3.92	No = 3.63	No = 3.33	No = 2.85	No = 4.40
	t = .332	t = -1.293	t = -.162	t = -.077	t = .411	t = .685	t = .233
	p = .740	p = .198	p = .871	p = .939	p = .682	p = .494	p = .816
	CI = -.206, .290	CI = -.866, .180	CI = -.700, .594	CI = -.681, .630	CI = -.411, .626	CI = -.281, .581	CI = -1.469, 1.863
**Received advice from doctors**	Yes = 3.22	Yes = 3.98	Yes = 3.78	Yes = 3.62	Yes = 3.27	Yes = 2.85	Yes = 4.35
	No = 3.20	No = 3.93	No = 3.93	No = 3.63	No = 3.34	No = 2.86	No = 4.42
	t = .190	t = .332	t = -.885	t = .031	t = -.525	t = -.112	t = -.160
	p = .851	p = .740	p = .377	p = .975	p = .600	p = .911	p = .873
	CI = -.169, .203	CI = -.228, .320	CI = -.488, .186	CI = -.432, .419	CI = -.342, .198	CI = -.238, .212	CI = -.940, .799
**Received advice from family or friends**	Yes = 3.30	Yes = 3.96	Yes = 4.12	Yes = 3.72	Yes = 3.44	Yes = 2.92	Yes = 5.02
	No = 3.16	No = 3.92	No = 3.81	No = 3.57	No = 3.28	No = 2.83	No = 4.08
	t = 3.421	t = .400	t = 2.888	t = 1.287	t = 1.808	t = 1.293	t = 3.439
	p = < .001*	p = .690	p = .004*	p = .200	p = .072	p = .198	p = < .001*
	CI = .060, .222	CI = -.141, .213	CI = .099, .526	CI = -.076, .363	CI = -.014, .332	CI = -.050, .240	CI = .405, 1.494
**Have seen media advertising about salt reduction**	Yes = 3.24	Yes = 3.99	Yes = 4.05	Yes = 3.63	Yes = 3.35	Yes = 2.89	Yes = 4.54
	No = 3.17	No = 3.87	No = 3.77	No = 3.62	No = 3.32	No = 2.82	No = 4.26
	t = 1.820	t = 1.459	t = 2.592	t = .130	t = .408	t = 1.029	t = 1.038
	p = .070	p = .146	p = .010*	p = .897	p = .684	p = .305	p = .301
	CI = -.006, .152	CI = -.044, .293	CI = .065, .483	CI = -.197, .225	CI = -.133, .202	CI = -.066, .211	CI = -.254, .818

Here, p = probability value; t = t-test; CI = confidence interval; and

* = statistically significant result.

A p-value of <0.05 is considered statistically significant. HT and NT represent hypertension and normotension respectively.

Participants who had received salt-reduction advice from their families or friends perceived a higher level of benefit if they took action to reduce their salt consumption (t = 2.888, p = .004, 95% CI: .099 –.526). However, they preferred to consume saltier foods than participants who had not received salt-reduction advice (t = 3.439, p = < .001, 95% CI: .405–1.494). Further, those participants who had seen media advertising about salt-reduction had an increased tendency to understand the perceived benefits of taking action (t = 2.592, p = .010, 95% CI: .065 –.483).

## Discussion

This aim of the present study was to explore the habitual dietary salt consumption among Chinese Australians, and factors that influenced their perceptions about reducing average salt consumption for the prevention of hypertension. The findings showed that only 2.7% (n = 5) participants measured the amount of salt added to their meals. Interestingly, 39.4% (n = 74) and 31.9% (n = 60) of participants reported that salt was added to their meals based on their experience and food taste respectively. Almost 90% (89.4%, n = 168) of participants indicated they have never received medical advice on salt restriction. Individuals’ salt-related knowledge had significant correlations with the perceptions of the severity of disease and health benefits of reducing salt consumption (p = .001 and < .001 respectively), but no significant correlations with their salty taste preferences. People with stronger salty taste preferences perceived a higher level of health threat than people with lighter salty taste preferences (p = .003).

To our knowledge, this is the first study of its kind in the context of Chinese residing in Western countries. The results of this study suggested that the design of self-management strategies should be culturally tailored to meet the needs of the Chinese community. The strategies should address the specific factors that facilitate and enable individuals to sustain dietary salt-related behavioural changes, especially at the primary health care level.

This study successfully recruited a unique group of participants, as a mix of Chinese people from different continents were included in this study. Our sample was younger (median = 34) than the median age of the Australian general population, which was 37 years in 2019 [42]. In line with our findings, an elevated BMI is a risk factor and predictor for hypertension [43]. Previous studies have found a strong link between obesity and high salt consumption [44, 45]. In this study, BMI had no statistical association with salty food preferences and the subscales related to perceptions of sustaining salt-related behavioural changes. With reference to the HBM, people are likely to initiate a health behavioural change if they perceive that they are at risk of suffering from hypertension and that their health would be significantly affected by hypertension [16]. However, participants with a higher BMI in this study had no significant elevated awareness of the negative health effects of excessive salt consumption or the perceived benefits of sustaining a health behavioural change. As a result, this group of people had no significant change in their behaviour regarding their preferences for salty food preferences.

### Habitual salt consumption behaviours

Our findings showed that participants in this study had many similarities in habitual salt consumption behaviours with their counterparts in China. Overall, 27.6% (n = 52) of participants reported they consumed pickles, soya sauce and bean paste, which were high in salt content, as additional seasoning at the dining table. This percentage of adding seasoning at the table was lower than the finding (31.4%) of a study conducted by Yang, Wang [46] in China but significantly lower than the finding (73%) from an Argentinian study [47].

Apart from Chinese people, many cultural and ethnic groups in the world, such as Argentines, Nepalese, Afghans and Vietnamese, had a similar habit of adding salt during home cooking [48, 49]. In Vietnam, salt use is part of the traditional diet, and culinary tradition was reported as a major barrier to initiating a salt-related behavioural change [48]. Using a salt-reduction tool such as a measuring spoon during home cooking is an implementation strategy recommended by local governments in China for many years, but unfortunately, the adoption level has remained low [50]. The present study affirmed that less than 3% of the participants (n = 5) measured their salt usage.

In the present study, personal preferences and practices acquired over a lifetime were the key drivers for salt consumption, findings which were similar to the results of two overseas studies [51, 52]. Motivation and willingness to sustain a salt-reduction behaviour were major challenges to many people’s habitual salt consumption habits. An Indonesian study found that food salt concentration significantly rebounded one week after a series of training and maintenance meeting sessions [53]. This further supports the notion that people’s habitual salt consumption behaviours may override their awareness of excessive salt consumption.

In Australia, salt-reduction initiatives have been established since 2009, and the Australian Government has committed to reducing the overall salt intake by a relative 30% by 2025 [54]. However, a systematic review in 2018 found that the mean salt consumption among Australians was 8.7g/day [55], a level almost twofold higher than the WHO guidelines of a maximum of 5g of salt per day. The effects of the current salt-reduction strategies, especially the reformulation of food products [56], were suboptimal. Greater effort could be made at the Federal and State Government levels in promoting and enforcing food reformulation. Health authorities and agents can promote changes to habitual food choices and preparation at major supermarkets and food outlets where the salt-reduction message can reach the wider population.

Considering that 75% of the Chinese Australians in this study were first-generation Australian residents [13], some of the salt-reduction strategies in Australia, such as the reformulated cereal-based products, may not apply to Chinese Australians’ regular food products. Nurses have an important role in patient education in all health care settings [57]. Nurses may consider tailoring culturally relevant salt-reduction strategies used overseas when providing education in Australia to this population group. For example, people in Singapore were encouraged to reduce their salt consumption by a small amount each day [58]. Chinese Australians with limited English and poor nutrition-label literacy skills would have difficulties in determining the salt content of food products [59]. Assisting Chinese Australians to select low-salt cultural food products may promote and empower their self-care ability to change their habitual salt-consumption behaviours.

### Salt-related health knowledge

This study revealed that the Chinese Australian participants had room for improvement in their salt-related knowledge and self-management of salt intake. Half of the participants were aware of the long-term health impact of a high-salt diet, including hypertension and renal disease. However, only 15% (n = 28) of participants knew that the WHO-recommended salt consumption for a healthy adult was 5g/day [5]. In comparison, a recent study in China found that over 78% of participants were aware of the daily recommended salt intake (5g/day), and 98% of participants were aware of the increased risk of hypertension associated with excessive dietary salt consumption [46]. The Chinese Australian group had significantly lower salt-related knowledge than their counterparts in China. Compared with other ethnic populations, our participants had a higher awareness of the daily recommended salt intake than study participants in Ethiopia and Northern India, where only 1.8% and 5% of participants respectively were aware of the daily recommended salt intake [60, 61]. However, over 87% of the participants in the Ethiopian study were aware of the increased risk of hypertension with a high salt intake. In comparison, our participants (n = 94, 50%) were less aware of this salt-related health issue.

Participants who had received salt-reduction advice from their families or friends had an increased awareness of the health benefits of salt-reduction. However, such awareness was not translated into action. This group of participants reported a higher salty food taste preferences than participants who had not received salt-reduction advice. Similar findings were found in Oman and the Republic of Moldova. Over 90% of respondents in both studies indicated awareness of salt-related health issues, but only 42.2% of respondents in Oman and 54% in the Republic of Moldova attempted to reduce their salt intake [62, 63]. This indicates that nurses and health care services may need to have a more specific focus on practical strategies such as salt alternatives, culturally relevant recipes, and shopping for reduced-salt products to promote the uptake of a low-salt diet at the population level.

Generally speaking, knowing the recommended maximum daily salt intake level is an essential part of promoting salt-reduction behaviours, and people who know the recommended daily salt intake are more likely to reduce salt consumption [64]. People who do not know the recommended daily salt limit may perceive that their salt intake is within the normal range but in fact, their daily intake may be over the recommended amount without their knowledge. This suggests that the Chinese Australians who have less than adequate salt-related knowledge, especially about the recommended daily salt limit, may be less aware of their dietary salt consumption and food choices.

In a literature review conducted by Chan et al. in 2022 [18] found that adequate salt-related health education had a positive influence on dietary behavioural changes within Chinese population groups. This finding was inconsistent with the present study. We found that salt-related health knowledge alone had no correlations with the perceived barriers and likelihood of taking action to reduce their health risks in this study. It is important to acknowledge that there was no statistical difference between participants with normotension and hypertension in terms of their salt-reduction knowledge and salty food taste preferences in the present study. In other words, Chinese Australians had inadequate awareness of the negative impact of dietary salt on humans. Language barriers and cultural differences may reduce Chinese Australians’ understanding of salt-reduction education and strategies designed for the general population. The voices of ethnic minority groups in campaigns to increase public awareness of salt-reduction are under-represented in Australia. Perhaps future health education strategies should not only be focused on improving salt-related knowledge, but also practice-based such as culturally relevant low-salt cooking recipes.

### Factors that influence Chinese Australians’ perceptions about sustaining salt-related behavioural changes

This study found that the factors influencing perceptions about sustaining salt-related behavioural changes included gender (men), single relationship status, BMI, salt-related knowledge, medical advice, and habitual salt consumption behaviours. Men in this study reported that they experienced a significantly higher level of barriers (t = 3.111, p = .002, 95% CI: .083 –.370) to reducing salt consumption than women. One explanation is that women are often the main cooks at home [46]. As a result, men have less power to control the amount of salt added during cooking. Another possible explanation is that with reference to the HBM, this group of people did not perceive the health benefits to be greater than the barriers (at an acceptable cost), and that they were capable (self-efficacy) of making the change [16]. So, they perceived a higher level of barriers and continued their high-salt dietary practice.

In our study, in general, single people preferred a higher amount of salt in food and experienced higher barriers to sustain dietary habit changes than married people. This may be associated with the social phenomenon that single adults more often eat out, consuming fast food or purchasing take-away food or food delivery rather than cooking at home [46]. Due to the aging population and economic changes, single-person households are the fastest growing demographic segment [65]. High-salt diets in single-person households may soon become a health concern in the Chinese Australian community. Future salt-reduction campaigns should pay more attention to this group of people. Their lifestyle and attitudes toward salt-reduction may not be the same as in the households of couples. The expectations of single people about using salt-reduction strategies needs further investigation.

The results of the current study revealed that there was no correlation between age and the perception of high salt intake (*r* = .061; p = .403) as a health threat. Previous studies have found that older people are often at a higher risk of suffering from cardiovascular disease [2, 66] and believed high-salt intake was a significant health risk [67]. These variations may be associated with the limited availability of culturally and linguistically appropriate health education for the older generations (predominantly the first generation of migrants), resulting in their not recognising the impact of salt on humans [68]. Therefore, future public health education should also consider the cultural relevance, linguistic factors and health literacy skills of older Chinese Australians.

It is worth noting that our findings showed that only 11% (n = 20) of participants had received medical advice about salt-reduction. This suggests that there is room for improvement in the primary prevention of salt-related hypertension. In line with our findings, personal preferences and practices acquired over a lifetime were the key drivers and facilitating factors for salt consumption [51, 52]. In this study, many participants reported that their salt usage was based on their experience or preferred taste, leading to suboptimal control of salt consumption. Lack of simple measurement methods to determine the salt content in foods [68] may be a confounding factor for the link between salt-related knowledge and perceptions of sustaining salt-related behavioural changes. This may result in suboptimal adherence to salt-reduction strategies. This relevant and important factor needs further investigation due to the limitation of the current study design.

The present study found that there were correlations among the salt-related health knowledge, perceived severity of disease and susceptibility, and benefits of taking action including using a measuring spoon to restrict salt consumption (*r* = .232, p = .001; *r* = .251, p = .001; *r* = .189, p = .009 respectively). With reference to the HBM, the perceptions of the health benefits and severity of disease could facilitate health behavioural changes [16, 17]. In this study, we extended the results of a previous study by Ma in 2017 [69]. Our findings supported that salt-related health knowledge should also be considered as a predictor and key factor affecting individuals’ salt consumption behaviours. However, there was a missing link between the knowledge of high dietary salt consumption on health and health behavioural changes for the prevention of hypertension.

In summary, the present study has extended our knowledge of the salt consumption behaviours and the factors that influenced the behavioural changes for the prevention of hypertension in Chinese Australian population. A key finding of this study was that single young Chinese Australian men with stronger salty taste preferences were the most important target group for salt-reduction interventions. Our findings show that salt-related health knowledge had significant correlations with perceived severity and susceptibility of disease. However, there were no correlations among the salt-related health knowledge, likelihood of following the recommended salt reduction interventions or perceived health threats and barriers to reducing salt consumption. Perhaps, the internal and external prompts that would trigger courses of action were missing.

The opportunity presents itself that nurse-led salt-reduction champions should be practice-based and culturally relevant to the Chinese Australian population as Chinese dietary practices are different from mainstream dietary practices. The culturally relevant practice-based strategies such as low-salt food choices and cooking methods may facilitate the awareness and interest of reducing salt consumption at the individual and community levels. Given that nurses have more direct patient care contact time with their patients than other health care providers, nurses can play a key role in identifying and refining existing health education programs to address individuals’ high dietary salt consumption problems.

### Limitations

The cross-sectional study design is unable to investigate longitudinal changes regarding the perceptions of and barriers to reducing salt consumption for hypertension prevention over time. Second, the questionnaire was fairly lengthy. Participants might drop out before they approached the end of the questionnaire. However, the response and dropout rates were 58.3% and 17.2% respectively. Two recent systematic reviews in 2020 and 2022 found that the overall response and dropout rates of mobile app- or internet-based survey were 44.1% [70] and 43% [71] respectively. So, the response and dropout rates of the present study were acceptable. In section 3 of the questionnaire, participants might have under- or over-reported their perceptions about the perceived threats of a high-salt diet, the benefits of preventive strategies and the barriers to reducing salt consumption. Third, selection bias might have occurred because almost 99% of the participants (n = 186, 98.9%) completed the questionnaire online. People with lower levels of digital literacy may be under-represented in this study.

## Conclusion

This study presented new insights into Chinese Australians’ dietary salt-related practice and knowledge, and the factors influencing their perceptions about sustaining salt-related behavioural changes, thus affecting their adherence to guidelines for reducing salt consumption for the prevention of hypertension. The knowledge generated from this Australian study demonstrated that the level of educational exposure to the health benefits of salt-reduction alone had no significant positive influence on dietary salt-related behavioural changes. Individuals with stronger salty taste preferences perceived a higher level of health treat, but lesser likelihood of following the recommended salt reduction interventions than individuals with lighter salty taste preferences. We recommend that the primary preventive health education should consider the cultural appropriateness of Chinese dietary practice and pay greater attention to facilitate the translation of salt-related health knowledge to action at the individual and community levels. Chinese dietary practices are different from mainstream dietary practices in Australia. Chinese Australians may appreciate practice-based strategies that can assist them to overcome the specific factors that make salt-reduction practices difficult to achieve.

## Supporting information

S1 Checklist STROBE checklist. (DOCX)

S1 Data Minimal data set. (XLSX)

## References

[1] Bi Z, Liang X, Xu A, Wang L, Shi X, Zhao W, et al. Hypertension prevalence, awareness, treatment, and control and sodium intake in Shandong Province, China: Baseline results from Shandong–Ministry of Health Action on Salt Reduction and Hypertension (SMASH), 2011. Prev Chronic Dis. 2014;11:E88. doi: 10.5888/pcd11.130423 24854239 PMC4032056

[2] Mente A, O’Donnell M, Rangarajan S, Dagenais G, Lear S, McQueen M, et al. Associations of urinary sodium excretion with cardiovascular events in individuals with and without hypertension: a pooled analysis of data from four studies. Lancet. 2016;388(10043):465–75. doi: 10.1016/S0140-6736(16)30467-6 27216139

[3] World Health Organization. A global brief on hypertension: Silent killer, global publichealth crisis [Internet]. Geneva (SZ): WHO; 2013 Jun 25 [https://www.who.int/publications/i/item/a-global-brief-on-hypertension-silent-killer-global-public-health-crisis-world-health-day-2013.

[4] World Health Organization. Salt reduction [Internet]. Geneva (SZ): WHO; 2020 Apr 29 [http://www.who.int/mediacentre/factsheets/fs393/en/.

[5] World Health Organization. Guideline: Sodium intake for adults and children [Internet]. Geneva (SZ): WHO; 2012 Dec 25 [https://www.who.int/publications/i/item/9789241504836. 23658998

[6] Grimes CA, Khokhar D, Bolton KA, Trieu K, Potter J, Davidson C, et al. Salt-related knowledge, attitudes and behaviors (KABs) among Victorian adults following 22-months of a consumer awareness campaign. Nutrients. 2020;12(5):1216. doi: 10.3390/nu12051216 32357458 PMC7282017

[7] Cappuccio FP, Chen J, Donfrancesco C, Palmieri L, Ippolito R, Vanuzzo D, et al. Geographic and socioeconomic variation of sodium and potassium intake in Italy: Results from the MINISAL-GIRCSI programme. BMJ Open. 2015;5(9):e007467. doi: 10.1136/bmjopen-2014-007467 26359282 PMC4577927

[8] Mukaneeva D, Kontsevaya A, Balanova Y, Khudyakov M, Drapkina O. Modelling the impact of compliance with WHO salt recommendations on cardiovascular mortality in Russia. Eur J Prev Cardiol. 2021;28(Suppl 1):i431. doi: 10.1093/eurjpc/zwab061.444

[9] Zhang P, He FJ, Li Y, Li C, Wu J, Ma J, et al. Reducing salt intake in China with "Action on Salt China" (ASC): protocol for campaigns and randomized controlled trials. JMIR Res Protoc. 2020;9(4):e15933. doi: 10.2196/15933 32271155 PMC7180507

[10] Bhat S, Marklund M, Henry ME, Appel LJ, Croft KD, Neal B, et al. A systematic review of the sources of dietary salt around the world. Adv Nutr. 2020;11(3):677–86. doi: 10.1093/advances/nmz134 31904809 PMC7231587

[11] Australian Bureau of Statistics. Migration, Australia [Internet]. Canberra (AU): Australian Bureau of Statistics; 2021 Apr 23 [https://www.abs.gov.au/statistics/people/population/migration-australia/latest-release#:~:text=In%202020%2C%20there%20were%20over,in%20Australia’s%20population%20in%202020.

[12] Australian Bureau of Statistics. Profiles of health, Australia 2011–2013 Cat.4338.0 [Internet]. Canberra: Australian Bureau of Statistics; 2013 [http://www.abs.gov.au/ausstats/abs@.nsf/Lookup/by%20Subject/4338.0~2011-13~Main%20Features~Daily%20intake%20of%20fruit%20and%20vegetables~10009.

[13] Australian Bureau of Statistics. ABS reveals insights into Australia’s Chinese population on Chinese new year [Internet]. Canberra (AU): Australian Bureau of Statistics; 2018 Feb 16 [https://www.abs.gov.au/AUSSTATS/abs@.nsf/mediareleasesbytitle/D8CAE4F74B82D446CA258235000F2BDE.

[14] Australian Bureau of Statistics. Ancestry [Internet]. Canberra: Australian Bureau of Statistics; 2016 [https://www.abs.gov.au/websitedbs/censushome.nsf/home/factsheetsa?opendocument&navpos=450.

[15] Wang Z, Chen Z, Zhang L, Wang X, Hao G, Zhang Z, et al. Status of hypertension in China. Circulation. 2018;137(22):2344–56. 29449338 10.1161/CIRCULATIONAHA.117.032380

[16] Rosenstock IM, Strecher VJ, Becker MH. Social learning theory and the health belief model. Health Educ Q. 1988;15(2):175–83. doi: 10.1177/109019818801500203 3378902

[17] Quaranta JE, Spencer GA. Using the health belief model to understand school nurse asthma management. J Sch Nurs. 2015;31(6):430–40. doi: 10.1177/1059840515601885 26324467

[18] Chan A, Chan SW-C, Khanam M, Kinsman L. Factors affecting reductions in dietary salt consumption in people of Chinese descent: An integrative review. J Adv Nurs. 2022;78(7):1919–37. doi: 10.1111/jan.15237 35384036 PMC9323495

[19] Liu Y, Shi M, Dolan J, He J. Sodium sensitivity of blood pressure in Chinese populations. J Hum Hypertens. 2020;34(2):94–107. doi: 10.1038/s41371-018-0152-0 30631129 PMC6698227

[20] Modesti PA, Marzotti I, Rapi S, Rogolino A, Cappuccio FP, Zhao D, et al. Daily urinary sodium and potassium excretion in Chinese first-generation migrants in Italy. Int J Cardiol. 2019;286:175–80. doi: 10.1016/j.ijcard.2018.12.029 30583922

[21] Rhodes K, Chan F, Prichard I, Coveney J, Ward P, Wilson C. Intergenerational transmission of dietary behaviours: a qualitative study of Anglo-Australian, Chinese-Australian and Italian-Australian three-generation families. Appetite. 2016:309–17. doi: 10.1016/j.appet.2016.04.036 27133550

[22] Lee M, Hu D, Bunney G, Gadegbeku CA, Edmundowicz D, Houser SR, et al. Health behavior practice among understudied Chinese and Filipino Americans with cardiometabolic diseases. Prev Med Rep. 2018;11:240–6. doi: 10.1016/j.pmedr.2018.06.004 30210996 PMC6129966

[23] Pasqualone A, Caponio F, Pagani MA, Summo C, Paradiso VM. Effect of salt reduction on quality and acceptability of durum wheat bread. Food Chem. 2019;289:575–81. doi: 10.1016/j.foodchem.2019.03.098 30955651

[24] Santos J, Webster J, Land M-A, Flood V, Chalmers J, Woodward M, et al. Dietary salt intake in the Australian population. Public Health Nutr. 2017;20(11):1887–94. doi: 10.1017/S1368980017000799 28511736 PMC10261523

[25] Fisher EB, Tang PY, Coufal MM, Liu Y, Jia W. Peer Support. In: Daaleman TP, Helton MR, editors. Chronic Illness Care: Principles and Practice. Midtown Manhattan (NY): Springer, Cham; c 2018. p. 133–46.

[26] Goulding T, Lindberg R, Russell CG. The affordability of a healthy and sustainable diet: an Australian case study. Nutr J. 2020;19(1):109. doi: 10.1186/s12937-020-00606-z 32998734 PMC7528590

[27] Chan A, Kinsman L, Chan SW-C. Psychometric testing of the Determinants of Salt-Restriction Behaviour Questionnaire in people of Chinese ancestry: a methodological study. BMC Nurs. 2022;21(1):339. doi: 10.1186/s12912-022-01124-5 36461075 PMC9717568

[28] von Elm E, Altman DG, Egger M, Pocock SJ, Gøtzsche PC, Vandenbroucke JP. The Strengthening the Reporting of Observational Studies in Epidemiology (STROBE) statement: guidelines for reporting observational studies. Ann Intern Med. 2007;147(8):573–7. doi: 10.7326/0003-4819-147-8-200710160-00010 17938396

[29] Australian Bureau of Statistics. 2016 Census [Internet]. Canberra (AU): Australian Bureau of Statistics; 2017 Apr 11 [https://www.abs.gov.au/websitedbs/censushome.nsf/home/2016.

[30] SurveyMonkey. Sample size calculator [Internet]. n.d. [https://www.surveymonkey.com/mp/sample-size-calculator/.

[31] Serdar CC, Cihan M, Yücel D, Serdar MA. Sample size, power and effect size revisited: simplified and practical approaches in pre-clinical, clinical and laboratory studies. Biochem Med. 2021;31(1):010502. doi: 10.11613/bm.2021.010502 33380887 PMC7745163

[32] Rice S, Traffimow D, Graves W, Stauble M. An expected value analysis of when to avoid type 1 and type 2 statistical errors in applied research. Proc Hum Factors Ergon Soc Annu Meet. 2013;57(1):1595–9. doi: 10.1177/1541931213571355

[33] Chen J, Liao Y, Li Z, Tian Y, Yang S, He C, et al. Determinants of salt-restriction-spoon using behavior in China: application of the health belief model. PLoS One. 2013;8(12):e83262. doi: 10.1371/journal.pone.0083262 24376675 PMC3869780

[34] Chen J, Tian Y, Liao Y, Yang S, Li Z, He C, et al. Salt-restriction-spoon improved the salt intake among residents in China. PLoS One. 2013;8(11):e78963. doi: 10.1371/journal.pone.0078963 24244395 PMC3823994

[35] Chen J, Liao YX, Li ZT, Tian Y, Yang SS, Tu DH, et al. [Analysis of the determinants of salt-restriction behavior among urban and rural residents in Beijing with Health Belief Model]. Beijing Da Xue Xue Bao. 2014;46(2):242–6. 24743814

[36] Abraham C, Sheeran P. The health belief model. In: Norman P, Conner M, editors. Predicting and changing health behaviour: Research and practice with social cognition models. 3rd ed. Maidenhead (GB): McGraw-Hill Education; 2015. p. 30–69.

[37] Alemayehu B, Kelbore AG, Alemayehu M, Adugna C, Bibo T, Megaze A, et al. Knowledge, attitude, and practice of the rural community about cutaneous leishmaniasis in Wolaita zone, southern Ethiopia. PLOS ONE. 2023;18(3):e0283582. doi: 10.1371/journal.pone.0283582 36976758 PMC10047512

[38] Kanji GK. 100 statistical tests. 3rd ed. London (GB): SAGE; 2006. 240 p.

[39] Madley-Dowd P, Hughes R, Tilling K, Heron J. The proportion of missing data should not be used to guide decisions on multiple imputation. J Clin Epidemiol. 2019;110:63–73. doi: 10.1016/j.jclinepi.2019.02.016 30878639 PMC6547017

[40] Lim JU, Lee JH, Kim JS, Hwang YI, Kim TH, Lim SY, et al. Comparison of World Health Organization and Asia-Pacific body mass index classifications in COPD patients. Int J Chron Obstruct Pulmon Dis. 2017;12:2465–75. doi: 10.2147/COPD.S141295 28860741 PMC5571887

[41] Australian Bureau of Statistics. Household income and wealth, Australia [Internet]. Canberra (AU): Australian Bureau of Statistics; 2022 Apr 28 [https://www.abs.gov.au/statistics/economy/finance/household-income-and-wealth-australia/latest-release.

[42] Australian Bureau of Statistics. Australian demographic statistics, June 2019 [Internet]. Canberra (AU): Australian Bureau of Statistics; 2019 Dec 19 [https://www.abs.gov.au/AUSSTATS/abs@.nsf/Lookup/3101.0Main+Features1Jun%202019?OpenDocument.

[43] Yu ES, Hong K, Chun BC. Incidence and risk factors for progression from prehypertension to hypertension: a 12-year Korean cohort study. J Hypertens. 2020;38(9):1755–62. doi: 10.1097/HJH.0000000000002494 32398468

[44] Allison SJ. High salt intake as a driver of obesity. Nat Rev Nephrol. 2018;14(5):285. 29578210 10.1038/nrneph.2018.23

[45] Zhou L, Stamler J, Chan Q, Van Horn L, Daviglus ML, Dyer AR, et al. Salt intake and prevalence of overweight/obesity in Japan, China, the United Kingdom, and the United States: the INTERMAP Study. Am J Clin Nutr. 2019;110(1):34–40. doi: 10.1093/ajcn/nqz067 31111867 PMC6599742

[46] Yang Y, Wang J, Ma J, Shi W, Wu J. Comparison of salt-related knowledge and behaviors status of WeChat users between 2019 and 2020. Nutrients. 2021;13(7):2141. doi: 10.3390/nu13072141 34206633 PMC8308297

[47] Elorriaga N, Gutierrez L, Romero IB, Moyano DL, Poggio R, Calandrelli M, et al. Collecting evidence to inform salt reduction policies in Argentina: identifying sources of sodium intake in adults from a population-based sample. Nutrients. 2017;9(9):964. doi: 10.3390/nu9090964 28858263 PMC5622724

[48] Webster J, Santos JA, Hogendorf M, Trieu K, Rosewarne E, McKenzie B, et al. Implementing effective salt reduction programs and policies in low- and middle-income countries: learning from retrospective policy analysis in Argentina, Mongolia, South Africa and Vietnam. Public Health Nutr. 2022;25(3):805–16. doi: 10.1017/S136898002100344X 34384514 PMC9991649

[49] Ghimire K, Mishra SR, Satheesh G, Neupane D, Sharma A, Panda R, et al. Salt intake and salt-reduction strategies in South Asia: from evidence to action. J Clin Hypertens. 2021;23(10):1815–29. doi: 10.1111/jch.14365 34498797 PMC8678780

[50] Chen S, Shan LC, Tao W, Lu T, Regan Á, Han H, et al. A survey of Chinese consumers’ knowledge, beliefs and behavioural intentions regarding salt intake and salt reduction. Public Health Nutr. 2020;23(8):1450–59. doi: 10.1017/S1368980019003689 31928552 PMC10200455

[51] Leong CS-F, Forde CG, T Siew Ling, Henry CJ. Taste perception and diet in people of Chinese ancestry. Asia Pac J Clin Nutr. 2018;27(2):478–86. doi: 10.6133/apjcn.052017.08 29384339

[52] Zou P. Facilitators and barriers to healthy eating in aged Chinese Canadians with hypertension: a qualitative exploration. Nutrients. 2019;11(1):111. doi: 10.3390/nu11010111 30626018 PMC6357039

[53] Irwan AM, Kato M, Kitaoka K, Ueno E, Tsujiguchi H, Shogenji M. Development of the salt-reduction and efficacy-maintenance program in Indonesia. Nurs Health Sci. 2016;18(4):519–32. doi: 10.1111/nhs.12305 27687887

[54] Lindberg R, Nichols T, Yam C. The healthy eating agenda in Australia. Is salt a priority for manufacturers? Nutrients. 2017;9(8):881. doi: 10.3390/nu9080881 28809812 PMC5579674

[55] Land M-A, Neal BC, Johnson C, Nowson CA, Margerison C, Petersen KS. Salt consumption by Australian adults: a systematic review and meta-analysis. Med J Aust. 2018;208(2):75–81. doi: 10.5694/mja17.00394 29385968

[56] Gressier M, Sassi F, Frost G. Contribution of reformulation, product renewal, and changes in consumer behavior to the reduction of salt intakes in the UK population between 2008/2009 and 2016/2017. Am J Clin Nutr. 2021;114(3):1092–9. doi: 10.1093/ajcn/nqab130 33963735 PMC8408870

[57] Bergh AL, Friberg F, Persson E, Dahlborg-Lyckhage E. Registered nurses’ patient education in everyday primary care practice: managers’ discourses. Glob Qual Nurs Res. 2015;2:1–12. doi: 10.1177/2333393615599168 28462314 PMC5342863

[58] Tan KW, Quaye SED, Koo JR, Lim JT, Cook AR, Dickens BL. Assessing the impact of salt reduction initiatives on the chronic disease burden of Singapore. Nutrients. 2021;13(4):1171. doi: 10.3390/nu13041171 33916184 PMC8065991

[59] Law QPS, Yau AHY, Chung JWY. Chinese adults’ nutrition label literacy in Hong Kong: implications for nurses. Nurs Health Sci. 2019;21(2):171–7. doi: 10.1111/nhs.12575 30345724

[60] Aparna P, Salve HR, Anand K, Ramakrishnan L, Gupta SK, Nongkynrih B. Knowledge and behaviors related to dietary salt and sources of dietary sodium in north India. J Family Med Prim Care. 2019;8(3):846–52. doi: 10.4103/jfmpc.jfmpc_49_19 31041212 PMC6482771

[61] Semira Mitiku S, Endris BS, Nagasa B, A Genet, G Seifu Hagos. Dietary sodium and potassium intake: knowledge, attitude and behaviour towards dietary salt intake among adults in Addis Ababa, Ethiopia. Public Health Nutr. 2021;24(11):3451–9. doi: 10.1017/S1368980020003663 33106202 PMC10195378

[62] Al-Riyami H, Al-Abdulsalam Q, Al-Khayari A, Al-Mushrafi H, Al-Alawi Z, Al-Hashmi K, et al. Awareness of the dangers of high salt intake among the urban Omani population. Sultan Qaboos Univ Med J. 2020;20(3):e352–e6. doi: 10.18295/squmj.2020.20.03.016 33110652 PMC7574804

[63] Chirsanova A, Sturza R, Capcanari T, Deseatnicova O, Boistean A, Vladislav R. Consumer behavior related to salt intake in the Republic of Moldova. J Soc Sci. 2020;III:101–10. doi: 10.5281/zenodo.4296387

[64] Dewey G, Wickramasekaran RN, Kuo T, Robles B. Does sodium knowledge affect dietary choices and health behaviors? Results from a survey of Los Angeles county residents. Prev Chronic Dis. 2017;14:E120–E. doi: 10.5888/pcd14.170117 29166247 PMC5703653

[65] Cho M, Bonn MA, Li J. Differences in perceptions about food delivery apps between single-person and multi-person households. Int J Hosp Manag. 2019;77:108–16. doi: 10.1016/j.ijhm.2018.06.019

[66] Jaul E, Barron J. Age-related diseases and clinical and public health implications for the 85 years old and over population. Front Public Health. 2017;5(335):1–7. doi: 10.3389/fpubh.2017.00335 29312916 PMC5732407

[67] Jing Z, Tao W, Hongling C, Xiangxian F, Jingpu S, Ruijuan Z, et al. Salt intake belief, knowledge, and behavior: a cross-sectional study of older rural Chinese adults. Medicine. 2016;95(31):1–5. doi: 10.1097/MD.0000000000004404 27495056 PMC4979810

[68] Michael V, You YX, Shahar S, Manaf ZA, Haron H, Shahrir SN, et al. Barriers, enablers, and perceptions on dietary salt reduction in the out-of-home sectors: a scoping review. Int J Environ Res Public Health. 2021;18(15):8099. doi: 10.3390/ijerph18158099 34360392 PMC8345971

[69] Ma C. An investigation of factors influencing self-care behaviors in young and middle-aged adults with hypertension based on a health belief model. Heart Lung. 2018;47(2):136–41. doi: 10.1016/j.hrtlng.2017.12.001 29395265

[70] Wu M-J, Zhao K, Fils-Aime F. Response rates of online surveys in published research: A meta-analysis. Computers in Human Behavior Reports. 2022;7:100206. doi: 10.1016/j.chbr.2022.100206

[71] Meyerowitz-Katz G, Ravi S, Arnolda L, Feng X, Maberly G, Astell-Burt T. Rates of Attrition and Dropout in App-Based Interventions for Chronic Disease: Systematic Review and Meta-Analysis. Journal of medical Internet research. 2020;22(9):e20283–e. doi: 10.2196/20283 32990635 PMC7556375

